# Impact of sirtuin-1 expression on H3K56 acetylation and oxidative stress: a double-blind randomized controlled trial with resveratrol supplementation

**DOI:** 10.1007/s00592-017-1097-4

**Published:** 2018-01-12

**Authors:** Simona Bo, Gabriele Togliatto, Roberto Gambino, Valentina Ponzo, Giusy Lombardo, Rosalba Rosato, Maurizio Cassader, Maria Felice Brizzi

**Affiliations:** 1 Department of Medical Sciences, University of Turin, Corso Dogliotti 14, 10126 Turin, Italy; 2 Department of Psychology, University of Turin, Turin, Italy

**Keywords:** Resveratrol, Epigenetics, PBMC, Sirtuin-1

## Abstract

**Aims:**

Sirtuin-1 (SIRT-1) down-regulation in type 2 diabetes mellitus (T2DM) has been associated with epigenetic markers of oxidative stress. We herein aim to evaluate whether an increase in SIRT-1 expression affects histone 3 acetylation at the 56 lysine residue (H3K56ac) in T2DM patients randomly selected to receive either resveratrol (40 mg or 500 mg) or a placebo for 6 months. The primary outcome is changes in the H3K56ac level by variation in SIRT-1 expression and the secondary outcome is the evidence of association between SIRT-1 level, antioxidant markers (TAS), and metabolic variables.

**Methods and results:**

At baseline, peripheral blood mononuclear cell H3K56ac values among the SIRT-1 tertiles did not differ. At trial end, SIRT-1 levels were significantly higher in patients receiving 500 mg resveratrol. At follow-up, patients were divided into tertiles of delta (trial end minus baseline) SIRT-1 value. Significant reductions in H3K56ac and body fat percentage were found in the highest tertile as were increased TAS levels. A multiple logistic regression model showed that the highest delta SIRT-1 tertile was inversely associated with variations in H3K56ac (OR = 0.66; 95% CI 0.44–0.99), TAS (OR = 1.01; 95% CI 1.00–1.02), and body fat percentage (OR = 0.75; 95% CI 0.58–0.96).

**Conclusions:**

We provide new knowledge on H3K56ac and SIRT-1 association in T2DM. These data suggest that boosting SIRT-1 expression/activation may impact redox homeostasis in these patients.

*ClinicalTrials.gov Identifier* NCT02244879.

## Introduction

Chronic diseases, including obesity and diabetes mellitus (T2DM), correlate with redox homeostasis imbalance. Mitochondrial reactive oxygen species (ROS) generation is considered the major regulator of hyperglycemia-mediated epigenetic changes, including histone (H) posttranslational modifications [1, 2]. In particular, H3K terminal tail acetylation largely results in gene transcription after genotoxic damage [3, 4]. Lo et al. [5] have recently shown that the highest H3 acetylation level at the 56 lysine residue (H3K56ac) is associated with proteins and transcription factors involved in T2DM signaling pathways in half of the mature adipocyte genome. H3K56 is deacetylated by the class III histone deacetylase (HDAC) sirtuins [3, 4]. One sirtuin family member, SIRT-1, was found persistently down-regulated in T2DM patients [6] and has been associated with mitochondrial ROS accumulation and increased p53 acetylation (p53ac) levels [7, 8]. Little data on the association between SIRT-1 and H3K56ac have been provided [3, 4, 9].

Resveratrol, 3,5,4′-trihydroxy-*trans*-stilbene, activates SIRT-1 and is a ROS scavenger [10]. Its benefits in human disease are highly controversial since not all the beneficial properties described in preclinical studies have been confirmed by clinical trials [11, 12], and concerns about its favorable effects were recently raised by systematic reviews and position statements [13–15]. We recently failed to detect any metabolic benefits of resveratrol in T2DM patients without vascular complications [16].

We therefore aimed to evaluate whether variations of SIRT-1 expression were associated with changes in H3K56ac and p53ac levels, and/or with modifications of oxidative stress markers, inflammatory or metabolic variables in T2DM patients. Peripheral blood mononuclear cells (PBMCs) were used as surrogates to evaluate SIRT-1, H3K56ac, and p53ac content. Unexpectedly, not all patients receiving resveratrol showed increased SIRT-1 expression/activation. Data were therefore analyzed by dividing participants according to increased SIRT-1 value, regardless of resveratrol treatment.

## Methods

### Study design and intervention

This was an observational study nested on a randomized controlled trial, which has previously been described [16, 17] and was registered at: www.clinicaltrials (Identifier: NCT02244879). Briefly, 192 T2DM patients were recruited from the Diabetic Clinic of the University of Turin from October 2013 through February 2016.

The inclusion criteria were: T2DM, age ≥ 40 years, body mass index (BMI) < 35 kg/m^2^, patients on diet and/or hypoglycemic agents other than insulin. Exclusion criteria were: treatment with any antioxidant substance, treatment with insulin, anticoagulants, steroids, or anti-inflammatory drugs different from aspirin, alcohol or substance abuse, uncompensated diabetes, liver or kidney diseases, presence of diabetes-related chronic complications, cardiovascular events or revascularization procedures in the previous 4-weeks, any severe chronic or life-threatening diseases, pregnancy, allergy to peanuts, grapes, wine, mulberries.

All patients recruited from March 2014 until February 2016 (*n* = 128) were enrolled for this study. All procedures were in agreement with the principles of the Helsinki Declaration; the study protocol was approved by the local ethics committee. All participants provided written informed consent.

Patients were randomized to one capsule/day of resveratrol 500 mg/day (Resv500 arm), one capsule/day of resveratrol 40 mg/day (Resv40 arm), and one capsule/day of placebo (totally inert microcellulose) (Placebo arm), respectively, for 6 months. Biotivia Bioceuticals (International SrL, Italy) prepared all the capsules, which were identical in size, shape, color, and taste. High-pressure liquid chromatography analyses revealed 99.7 and 97.9% trans-resveratrol purity in 500 mg and 40 mg capsules, respectively, and no resveratrol content in placebo capsules.

Patients and researchers who dispensed the capsules, collected data, and were involved in all measurements were blinded to the bottle content.

Participants were allocated as follows: 43, 43, and 42 in the Resv500, Resv40, and Placebo arms, respectively. All patients took medication in the morning and maintained their habitual lifestyle, the diet given by the Diabetic Clinic, their current hypoglycemic treatment and were instructed to abstain from nutritional supplements and significant amounts of resveratrol-rich foods and beverages [12, 16]. Compliance with the study protocol was monitored with monthly phone calls and pill counting.

Patients were stratified by acetylsalicylic acid use and glycated hemoglobin (HbA1c) levels (cut-point 7%), according to a computer-generated randomization sequence [16, 17].

### Outcomes

#### *Primary outcome*

Association between changes in SIRT-1 level and variation in H3K56ac value.

#### *Secondary outcomes*

Association between changes in SIRT-1 level and variation in p53ac, oxidative stress markers (total antioxidant status: TAS), anthropometric, metabolic, and inflammatory variables.

### Measurements

Body weight and height were measured with light clothes and no shoes. Waist circumference was measured at the narrowest level by a plastic tape meter. Body composition was determined by dual X-ray densitometry (QDR-4500 Hologic Inc., Bedford, MA, USA), using whole-body absorptiometry software. Arterial blood pressure values were measured twice from the left arm, in a sitting position, after at least 10-min rest, with a mercury sphygmomanometer with appropriate cuff sizes (ERKA Perfect-Aneroid, Germany). The homeostasis model assessment-insulin resistance (HOMA-IR) was calculated according to the published algorithm.

### Blood sample analysis

Blood samples were freshly collected from each participant at baseline and at trial end. The laboratory measurements were blindly performed at the Laboratories of the Department of Medical Sciences, University of Turin.

The human PTX3 was measured by a ready-to-use solid-phase enzyme-linked immunosorbent assay based on the sandwich principle (Hycult biotech, Uden, The Netherlands). The intra-assay and inter-assay CVs were 3.6–3.8 and 4.1–4.9%, respectively. TAS measurement was performed with a colorimetric assay (ImAnOx TAS Kit, Immundiagnostik AG Bensheim, Germany), with intra-assay and inter-assay CVs of 2.0–4.0 and 2.6–3.9%, respectively.

Serum C-reactive protein (CRP) values were determined using a high-sensitivity latex agglutination assay on HITACHI 911 Analyzer (Sentinel Ch., Milan, Italy). The intra-assay and inter-assay CVs were 0.8–1.3 and 1.0–1.5%, respectively. Interleukin-6 (IL-6) circulating concentrations were measured by a quantitative sandwich enzyme immunoassay technique (R&D System, Minneapolis, MN, USA) with an intra-assay CV of 6.9% and an inter-assay CV of 7.2%.

Serum glucose was measured by the glucose oxidase method (Sentinel Ch., Milan) with an intra-assay CV of 1.1% and an inter-assay CV of 2.3%. Triglycerides and cholesterol were assayed by enzymatic colorimetric assays (Sentinel Ch., Milan) with an intra-assay CV of 3.0% and an inter-assay CV of 3.5% for triglycerides and with an intra-assay CV of 2.2% and an inter-assay CV of 3.4% for cholesterol. HDL-cholesterol was determined by enzymatic colorimetric assay after precipitation of LDL and VLDL fractions using heparin–MnCl2 solution and centrifugation at 4 °C, and it had an intra-assay CV of 2.5% and an inter-assay CV of 4.1%. Free fatty acid (FFA) values were assayed by an enzymatic colorimetric method (RANDOX, UK).

Alanine aminotransferase (ALT) and ɣ-glutamyl-transferase (GGT) were measured by a kinetic determination (Sentinel Ch., Milan) according to the IFCC recommendations. Insulin was measured by a biotin labeled antibody based sandwich enzyme immunoassay (LDN, Germany). The kit had a sensitivity of less than 1.8 μU/ml and a range of 0–100 μU/ml. The intra-assay and inter-assay CVs were 1.8–2.6 and 2.9–6.0%, respectively. Glycated hemoglobin (Hba1c) was determined with a latex-based method (Sentinel Ch., Milan). The intra-assay and inter-assay CVs were 1.1–1.5 and 1.1–1.6%, respectively. Adiponectin was measured by sandwich enzyme-linked immunosorbent assays (BioVendor, Brno, Czech Republic). The kit has a sensitivity of 470 ng/ml and a range of 5000 to 150,000 ng/ml. The intra- and inter-assay CVs were 4.1 and 6.9%, respectively. Uric acid was assayed by uricase-based enzymatic colorimetric assays (Sentinel Ch., Milan) with an intra-assay CV of 2.1% and an inter-assay CV of 1.7%.

#### Peripheral blood mononuclear cell (PBMC) preparation

Blood sample (14 mL) was freshly collected from each patient participating in the randomized controlled trial (RCT) at baseline and at the end of treatment. PBMCs were isolated by Ficoll-Paque (Histopaque 1077; Sigma-Aldrich) density gradient centrifugation (400 g for 30 min at RT), washed twice with saline phosphate buffer (PBS) (300 g for 10 min at 4 °C), and lysed using a lysis buffer for nuclear protein extraction.

#### Western blot analysis

PBMCs were lysed as previously described [18]. Proteins (50 µg) were subjected to SDS-PAGE, transferred into nitrocellulose membrane, blotted with the indicated antibodies, and processed. Densitometric analysis was used to calculate the differences in the fold induction of protein levels normalized to β-actin, H3, and p53 content. Twenty samples, randomly selected, were double analyzed, and similar results were obtained. Values are reported as relative amounts (ra). All experiments were blindly performed.

### Statistics

Within-group differences among the three arms were evaluated using the Wilcoxon matched-pairs test, while between-group differences were computed using the Kruskal–Wallis test.

Participants were divided according to tertiles of the baseline SIRT-1 values; the highest tertile (SIRT-1 ≥ 1.16ra) was compared with the other two. The differences between end-of-the-trial values and each variable’s baseline value (delta) were calculated. Delta SIRT-1 tertiles were then computed; analyzed variable delta values were compared with the highest tertile of delta SIRT-1 (≥ 0.66ra) and the other two.

Either the Student’s *t* test or the Mann–Whitney *U* test (non-normally distributed variables) was used to investigate differences in the variables between SIRT-1 value tertiles.

Associations between the highest tertile of delta SIRT-1 values and the median values of delta H3K56ac, delta TAS, and delta percent body fat were evaluated using a multiple logistic regression model, after adjustments for age, gender, and resveratrol treatment.

The power of the study, calculated by using the Wilcoxon test for delta H3K56ac mean values, was 0.82 with *α*-value = 0.05.

## Results

### Baseline expression of SIRT-1 and its targets, H3K56ac and p53ac

Mean ± SD and median (interquartile range) baseline values of SIRT-1 in T2DM patients were 1.04 ± 0.71 and 0.86 (0.93) ra. Table 1 shows patient characteristics, by SIRT-1 tertiles at baseline (the highest tertile was compared to the lowest two tertiles). We found that patients in the highest tertile showed higher HbA1c values. No differences in H3K56ac and p53ac values were detected in the two groups.

**Table 1:** Baseline characteristics of T2DM patients by SIRT-1 tertiles

Baseline variables	Lower tertiles (< 1.16 ra)	Highest tertile (≥ 1.16 ra)	P*
Number	86	42	
Males (%)	67.4	54.8	0.16
Age (years)	65.6 ± 7.3	64.0 ± 9.4	0.31
Weight (kg)	80.3 ± 12.5	83.8 ± 15.8	0.18
BMI (kg/m^2^)	29.1 ± 3.7	30.2 ± 3.6	0.13
Waist circumference (cm)	102.7 ± 9.7	105.5 ± 11.3	0.15
Percent body fat	33.5 ± 6.7	35.6 ± 7.3	0.10
Systolic blood pressure (mmHg)	132.1 ± 10.1	132.1 ± 8.4	0.98
Diastolic blood pressure (mmHg)	80.7 ± 8.2	80.4 ± 7.6	0.87
Fasting glucose (mg/dl)	138.8 ± 33.3	145.0 ± 43.5	0.37
Glycated hemoglobin (%) (mmol/mol)	6.75 ± 0.86	7.16 ± 1.34	0.038
	50.3 ± 9.4	54.7 ± 14.6	
Fasting C-peptide (nmol/l)	0.95 ± 0.44	1.05 ± 0.48	0.21
Fasting insulin (µU/ml)**	16.2 (11.0)	18.2 (10.6)	0.34
HOMA-IR (mmol/L × µU/ml)**	5.29 (4.03)	5.41 (4.02)	0.26
Total cholesterol (mg/dl)	176.6 ± 37.9	184.6 ± 40.9	0.28
Triglycerides (mg/dl)**	106.5 (86.0)	111.0 (77.0)	0.52
HDL-cholesterol (mg/dl)	46.7 ± 12.9	44.6 ± 10.2	0.38
LDL-cholesterol (mg/dl)	103.1 ± 34.3	112.9 ± 34.0	0.13
Free fatty acids (mmol/l)	0.66 ± 0.19	0.69 ± 0.19	0.40
Pentraxin-3 (ng/ml)**	0.81 ± 0.58	0.70 ± 0.39	0.29
Interleukin-6 (pg/ml)**	2.83 (2.38)	2.41 (1.43)	0.13
C-reactive protein (mg/dl)**	1.52 (2.47)	1.56 (3.67)	0.66
Adiponectin (ng/ml)**	7545.1 (4746.5)	7188.9 (5974.1)	0.72
Total antioxidant status (µmol/L)	294.2 ± 41.0	291.3 ± 41.4	0.71
Uric acid (mg/dl)	5.7 ± 1.4	5.4 ± 1.2	0.23
Alanine aminotransferase (U/l)	19.5 ± 9.6	18.7 ± 8.9	0.65
ɣ-glutamyl-transferase (U/l)**	23.0 (19.0)	25.0 (18.0)	0.83
H3K56ac (ra)**	0.88 (0.85)	0.96 (1.12)	0.39
p53ac (ra)	0.78 ± 0.53	0.90 ± 0.64	0.27

*BMI* body mass index, *HOMA-IR* homeostasis model assessment-insulin resistance, *ra* relative amount

*P obtained by Student’s *t* test or Chi-square, as appropriate

**Median (interquartile range); *P* obtained by Mann–Whitney *U* test

### Increased SIRT-1 expression correlates with a reduction in H3K56ac, but not p53ac

**Fig. 1:**
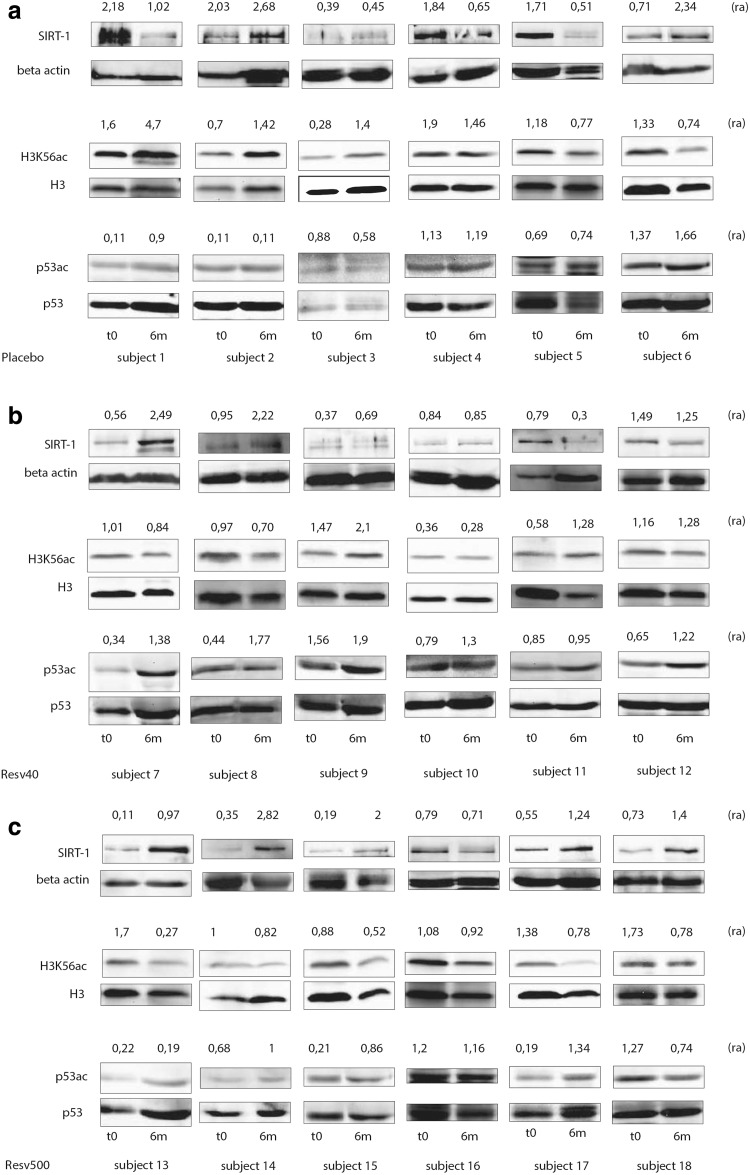
SIRT-1, H3K56ac, and p53ac protein expression in PBMCs recovered from T2DM patients submitted to resveratrol supplementation or placebo. **a** Cell extracts from T2DM patients’ PBMCs (subject 1–6) submitted to placebo were analyzed for SIRT-1, H3K56ac, and p53ac content at baseline (t0) and at the trial end (6 m). Protein levels were normalized to beta actin, H3 and p53 content, respectively. The values reported represent the relative amount (ra) of protein expression obtained by densitometric analysis. **b** Cell extracts from T2DM patients’ PBMCs (subject 7–12) submitted to resveratrol 40 mg/day supplementation (Resv40) were lysed and analyzed for SIRT-1, H3K56ac, and p53ac content at baseline (t0) and at the trial end (6 m). Protein levels were normalized to beta actin, H3 and p53 content, respectively. The values reported represent the relative amount (ra) of protein expression obtained by densitometric analysis. **c** Cell extracts from T2DM patients’ PBMCs (subject 13–18) submitted to resveratrol 500 mg/day supplementation (Resv500) were lysed and analyzed for SIRT-1, H3K56ac, and p53ac content at baseline (t0) and at the trial end (6 m). Protein levels were normalized to beta actin, H3, and p53 content, respectively. The values reported represent the relative amount (ra) of protein expression obtained by densitometric analysis

**Fig. 2:**
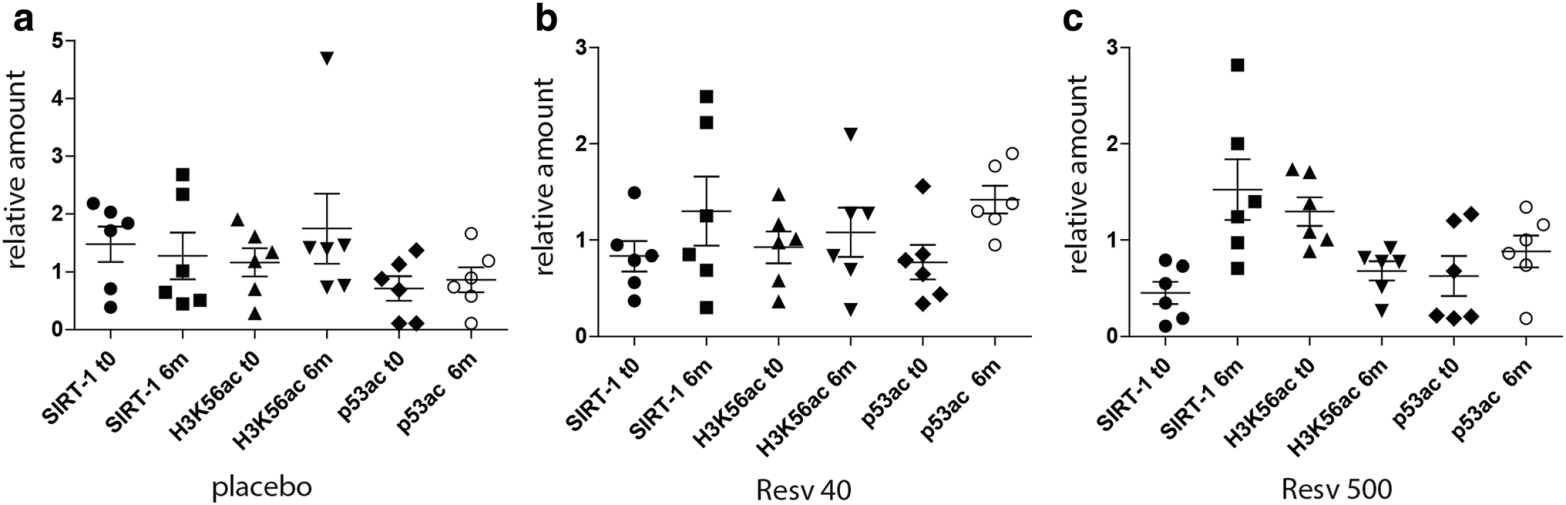
Relative amount of SIRT-1, H3K56ac, and p53ac protein expression in PBMCs recovered from T2DM patients submitted to resveratrol supplementation or placebo. **a** The value reported represents the relative amount of protein expression obtained by densitometric analysis of cell extract analyzed in Fig. 1a. Cell extract from T2DM patient’ PBMCs (subject 1–6) submitted to placebo was lysed and analyzed for SIRT-1, H3K56ac, and p53ac content at baseline (t0) and at trial end (6 m). Protein levels were normalized to beta actin, H3, and p53 content, respectively. **b** The value reported represents the relative amount of protein expression obtained by densitometric analysis of cell extract analyzed in Fig. 1b. Cell extract from T2DM patient’ PBMCs (subject 7–12) submitted to resveratrol 40 mg/day supplementation (Resv40) was lysed and analyzed for SIRT-1, H3K56ac, and p53ac content at baseline (t0) and at trial end (6 m). Protein levels were normalized to beta actin, H3, and p53 content, respectively. **c** The value reported represents the relative amount of protein expression obtained by densitometric analysis of cell extract analyzed in Fig. 1c. Cell extract from T2DM patient’ PBMCs (subject 13–18) submitted to resveratrol 500 mg/day supplementation (Resv500) was lysed and analyzed for SIRT-1, H3K56ac, and p53ac content at baseline (t0) and at trial end (6 m). Protein levels were normalized to beta actin, H3, and p53 content, respectively

The following dropouts occurred at follow-up: two (Placebo arm), two (Resv40 arm), one (Resv500 arm). At trial end, the mean ± SD and median (interquartile range) values of SIRT-1 were 1.37 ± 1.65 and 0.97 (1.23) ra. The mean delta SIRT-1 value was 0.36ra. The values of SIRT-1, by trial arm, are reported in Table 2. Within-group SIRT-1 values significantly increased at trial end in the Resv500 group (*p* < 0.001 by Wilcoxon test), and the delta values differed among the groups. Median H3K56ac and p53ac values did not differ among arms of treatment. The median delta values for each analyzed variable, by delta SIRT-1 tertiles, are shown in Table 3 (left side). A significantly higher proportion of Resv500 arm patients showed higher increases in SIRT-1 value. Patients in the highest delta SIRT-1 tertile displayed a significant decrease in H3K56ac. No significant differences in p53ac expression levels were detected. SIRT-1, H3K56ac, and p53ac expression levels for six patients in each arm are reported in Figs. 1 and 2.

**Table 2:** Median values of SIRT-1, H3K56ac, and P53ac, expressed as relative amounts, after treatment with resveratrol or placebo

	Placebo	Resv40	Resv500	*P*
*SIRT*-*1*				
Baseline	0.93 (1.00)	0.81 (1.01)	0.79 (0.79)	0.74
Trial end	0.79 (0.82)	0.90 (1.18)	1.16 (1.64)	0.14
Delta	− 0.16 (1.33)	0.01 (1.36)	0.45 (1.21)	0.046
*H3K56ac*				
Baseline	0.87 (1.06)	0.85 (1.02)	1.04 (0.96)	0.56
Trial end	1.09 (0.69)	1.16 (0.86)	1.27 (0.93)	0.90
Delta	0.34 (1.18)	0.12 (1.04)	0.03 (1.26)	0.70
*P53ac*				
Baseline	0.89 (0.76)	0.79 (0.85)	0.51 (1.01)	0.06
Trial end	0.81 (0.69)	1.06 (0.99)	0.93 (0.78)	0.25
Delta	0.06 (0.60)	0.22 (0.92)	0.19 (0.88)	0.94

Delta end of trial minus baseline value; median (interquartile range)

*P* values obtained by Kruskal–Wallis test

**Table 3:** Median value of deltas (end-of-trial value minus baseline value) for each of the analyzed variables by tertiles of delta SIRT-1 in all participants (left side) and after excluding individuals from the Placebo arm (right side)

	Lower tertiles (< 0.66 ra)	Highest tertile (≥ 0.66 ra)	P*	Lower tertiles	Highest tertile	P*
Number	83	40		50	33	
SIRT-1 (ra)	− 0.23 (0.73)	1.29 (0.91)	<0.001	− 0.14 (0.53)	1.28 (1.04)	<0.001
Placebo arm (%)	39.8	17.5		–	–	
Resv40 arm (%)	33.7	32.5		56.0	39.4	
Resv500 arm (%)	26.5	50.0	0.014	44.0	60.6	0.14
Males (%)	61.5	70.0	0.35	60.0	63.6	0.74
Weight (kg)	− 0.50 (2.50)	− 0.10 (3.75)	0.82	− 0.25 (3.00)	− 0.20 (4.00)	0.77
BMI (kg/m^2^)	0.00 (0.90)	− 0.057 (1.35)	0.82	0.00 (1.28)	− 0.09 (1.39)	0.43
Waist circumference (cm)	1.75 (6.75)	0.00 (5.00)	0.55	0.00 (4.50)	1.50 (6.50)	0.52
Percent body fat	0.20 (1.80)	− 0.60 (3.00)	0.018	0.00 (1.80)	− 0.45 (3.00)	0.13
Systolic blood pressure (mmHg)	0.00 (12.0)	0.00 (12.5)	0.71	0.00 (10.0)	0.00 (10.0)	0.41
Diastolic blood pressure (mmHg)	0.00 (10.0)	0.00 (15.0)	0.60	0.00 (12.0)	0.00 (10.0)	0.17
Fasting glucose (mg/dl)	2.00 (27.0)	− 2.00 (24.5)	0.32	2.00 (20.0)	1.00 (21.0)	0.28
Glycated hemoglobin (%) (mmol/mol)	0.30 (1.40)	0.25 (1.15)	0.20	0.35 (1.30)	0.20 (1.10)	0.22
	3.28 (15.3)	2.73 (12.6)		3.83 (14.2)	2.19 (12.0)	
Fasting C-peptide (nmol/l)	− 0.040 (0.43)	− 0.06 (0.47)	0.45	− 0.02 (0.47)	− 0.08 (0.47)	0.21
Fasting insulin (µU/ml)	2.89 (9.76)	0.32 (9.29)	0.26	2.89 (12.8)	1.81 (9.26)	0.28
HOMA-IR (mmol/L × µU/ml)	1.04 (3.75)	0.17 (3.39)	0.22	1.13 (5.03)	0.28 (2.94)	0.32
Total cholesterol (mg/dl)	− 7.50 (43.0)	− 1.00 (48.0)	0.31	− 4.00 (38.0)	− 3.00 (50.0)	0.76
Triglycerides (mg/dl)	13.00 (55.0)	12.50 (45.5)	0.66	16.0 (71.0)	14.0 (46.0)	0.40
HDL-cholesterol (mg/dl)	2.00 (12.0)	2.50 (10.0)	0.37	2.00 (12.0)	3.00 (11.0)	0.16
LDL-cholesterol (mg/dl)	− 6.50 (34.0)	− 4.50 (41.5)	0.48	− 3.50 (32.0)	− 3.00 (43.0)	0.95
FFA (mmol/l)	0.01 (0.04)	0.01 (0.04)	0.93	0.01 (0.06)	0.01 (0.04)	0.79
Pentraxin-3 (ng/ml)	0.04 (0.60)	0.08 (0.62)	0.82	0.13 (0.71)	0.00 (0.60)	0.36
Interleukin-6 (pg/ml)	0.19 (1.64)	− 0.27 (1.60)	0.16	0.32 (1.84)	− 0.23 (1.70)	0.33
C-reactive protein (mg/dl)	− 0.02 (2.51)	0.00 (2.22)	0.38	0.00 (2.63)	0.01 (2.22)	0.54
Adiponectin (ng/ml)	137.4 (3583.8)	653.0 (3555.4)	0.71	− 449.6 (3763.8)	463.3 (3217.0)	0.43
Total antioxidant status (µmol/L)	− 2.00 (62.0)	13.5 (72.0)	0.033	4.00 (70.0)	25.0 (66.0)	0.047
Uric acid (mg/dl)	0.00 (1.40)	0.30 (1.25)	0.32	0.05 (1.50)	0.30 (1.20)	0.44
ALT (U/l)	2.00 (11.0)	− 1.00 (12.5)	0.12	1.00 (13.0)	− 1.00 (14.0)	0.21
GGT (U/l)	1.00 (7.00)	0.00 (8.00)	0.68	2.00 (9.00)	0.00 (9.00)	0.29
H3K56ac (ra)	0.34 (0.95)	− 0.20 (1.40)	0.025	0.25 (1.04)	− 0.22 (1.37)	0.028
P53ac (ra)	0.10 (0.80)	0.32 (1.02)	0.24	0.18 (0.74)	0.40 (1.30)	0.50

Median (interquartile range) *P obtained by Mann–Whitney *U* test or Chi-square, as appropriate

*BMI* body mass index, *HOMA-IR* homeostasis model assessment-insulin resistance, *FFA* free fatty acids, *ALT* alanine aminotransferase, *GGT* ɣ-glutamyl-transferase, *ra* relative amount

The deltas of the different variables, by SIRT-1 variation, were calculated after excluding patients from the Placebo arm (Table 2, right side). Patients in the highest SIRT-1 value tertile still displayed a significant reduction in H3K56ac levels.

In a multiple logistic regression model, the highest delta SIRT-1 tertile was inversely associated with H3K56ac variation and directly related to resveratrol supplementation in all patients (Table 4). These associations were no longer statistically significant after excluding patients from the Placebo arm.

**Table 4:** Multiple logistic regression model. Associations between the highest tertile of delta SIRT-1 value and median value of delta H3K56ac (upper), median value of TAS (middle), and delta percentage body fat (lower) in all arms of the trial (left) or including only individuals submitted to resveratrol supplementation (right)

	All arms			Resveratrol arms		
	OR	95% CI	*P*	OR	95% CI	*P*
Delta H3K56ac	0.66	0.44–0.99	0.04	0.67	0.43–1.04	0.07
Age	1.05	0.99–1.10	0.10	1.02	0.96–1.08	0.58
Male gender	1.84	0.76–4.47	0.17	1.27	0.49–3.31	0.62
Treatment with resveratrol	3.58	1.29–9.90	0.01	–		
Delta TAS	1.01	1.00–1.02	0.04	1.01	0.99–1.02	0.05
Age	1.04	0.99–1.10	0.11	1.02	0.96–1.08	0.50
Male gender	2.02	0.83–4.95	0.12	1.43	0.54–3.79	0.47
Treatment with resveratrol	3.09	1.16–8.22	0.02	–		
Delta percent body fat	0.75	0.58–0.96	0.02	0.81	0.63–1.05	0.11
Age	1.05	0.98–1.11	0.14	1.02	0.95–1.08	0.75
Male gender	2.37	0.94–5.96	0.07	1.58	0.59–4.21	0.35
Treatment with resveratrol	3.65	1.36–9.81	0.01	–		

### Increased SIRT-1 expression was directly associated with total antioxidant status

Highest delta SIRT-1 tertile patients showed significant increments in TAS values (Table 3), and that were still evident after excluding Placebo arm individuals. The direct association between median delta SIRT-1 values and delta TAS was confirmed by multiple regression analyses (Table 4).

### Comparison between SIRT-1 expression and metabolic and inflammatory variables

Individuals in the highest SIRT-1 tertile showed a significant percentage body fat decrease (Table 3). This significant inverse relationship was confirmed using a multiple regression model (Table 4), but not after excluding the Placebo group.

No other metabolic variable was associated with changes in SIRT-1 expression. Similarly, no association between SIRT-1 change and inflammatory variables was found.

## Discussion

We herein report that, at 6-month follow-up, increased SIRT-1 expression was associated with significant H3K56ac content reduction and increased serum antioxidant activity in T2DM patients.

SIRT-1 is a nicotinamide adenine dinucleotide (NAD)-dependent deacetylase, involved in the deacetylation of histones and nonhistone proteins, including transcription factors, proteins and enzymes implicated in cell differentiation, cell survival, and metabolism [19, 20]. p53 was the first SIRT-1 target to be identified [21]. However, SIRT-1 also deacetylates H3K56, a crucial regulator of chromatin assembly/disassembly, which mainly marks transcriptionally active genes after genotoxic cues [3, 4]. More recently, genome-wide analysis demonstrated that highest levels of H3K56 acetylation are associated with diabetes-related genes [5]. Indeed, changes in histone acetylation have a central role in sustaining the long-lasting effects of hyperglycemia mechanistically defined as “metabolic memory” [22]. As a matter of fact, SIRT1 also controls mitochondrial function and biogenesis, and free radical formation, key cues associated with epigenome reprogramming in diabetes [23]. Resveratrol is one of the most active polyphenols to stimulate SIRT-1 activity [24], which is known to be reduced in T2DM patients [6]. Therefore, we aimed to investigate whether rescue of SIRT-1 expression/activation could influence diabetes-related oxidative stress via H3K56 deacetylation. Unexpectedly, we found that not all T2DM patients receiving resveratrol displayed increased SIRT-1 expression/activation. Nevertheless, the increase in SIRT-1 expression was associated with changes in H3K56ac.

PBMCs were used as surrogates to evaluate SIRT-1 expression, while p53 and H3K56 acetylation were used as markers of its activation. A significant and dose-dependent increase in SIRT-1 expression was found after resveratrol supplementation. However, not all resveratrol arm patients showed an increase in SIRT-1 value at trial end. Changes in lifestyle and/or dietary intake, such as resveratrol-rich foods and beverages during the trial, may have caused unpredictable environmental conditions which may have influenced SIRT-1 expression. Moreover, genetically determined variants of the individual response to resveratrol treatment may also be postulated. Finally, the possibility of SIRT-1 being a direct target of resveratrol has been questioned [25], while both SIRT-1-independent effects [26] and decreased SIRT-1 levels [27] have been reported after resveratrol treatment.

Particular attention has recently been dedicated to the identification of oxidative stress-induced epigenetic T2DM markers [8]. Indeed, increased HDAC3 activity in PBMCs from T2DM patients has been directly correlated with inflammation and insulin resistance, and negatively with SIRT-1 level [28]. Accordingly, HDAC activity suppression in embryos subjected to hyperglycemia-induced oxidative stress resulted in increased H3K56ac level [29]. H3K56ac has been linked to the activation of genes related to the unbalance of redox signals [3, 5, 9]. The inverse relationship between SIRT-1 level and H3K56ac is consistent with the original observation that antioxidant treatments reduced H3K56ac [3]. The significant correlation between increased serum antioxidant activity and SIRT-1/H3K56ac expression may be a proof of concept.

T2DM is characterized by low-grade chronic inflammation, which has been linked to low sirtuin levels [30]. A close connection between SIRT-1 and p53ac has been reported, primarily in diabetes-associated vascular dysfunction/inflammation [8, 30]. Long-lasting diabetes (median value: 8-y) is a relevant feature in our patients. An impaired adaptive SIRT-1-mediated counter-acting response to environmental cues may therefore be a possible explanation for the failure to find correlation between SIRT-1, p53ac, and inflammatory parameters. Alternatively, the low sample size may have masked small, but meaningful beneficial SIRT-1 effects on the inflammatory profile.

SIRT-1 controls the acetylation of transcriptional regulators involved in adipogenesis and fatty acid oxidation [31]. The anti-adipogenic effects of resveratrol have been demonstrated in preclinical models, while controversial results were found in humans [12, 13]. We found a significant reduction in body fat percentage in individuals with higher SIRT-1 expression. However, this effect was lost when placebo patients were excluded. These results suggest that SIRT-1 plays a role as an anti-adipogenic factor, while suggesting that different epigenetic mechanisms may be involved in SIRT-1-mediated anti-adipogenic effects.

SIRT-1 has also been shown to regulate glucose and lipid metabolism and therefore reduce cardiovascular risk factors [30, 31]. We failed to find any association between SIRT-1 expression and metabolic variables. Highest SIRT-1 tertile patients showed increased HbA1c values at baseline and, a worse, though not significantly different metabolic profile overall. Compensatory SIRT-1 activation might be hypothesized; otherwise, it may be considered a casual finding.

### Clinical implications

Although the physio-pathological mechanisms implicated are quite complex and multiple confounders may have biased our results, we herein provide evidence that boosting SIRT-1 expression translates into a significant reduction in H3K56ac, an epigenetic marker of oxidative stress-mediated damage, and into improvements in antioxidant activity. Understanding SIRT-1’s role in the in vivo regulation of human metabolism and health still remains a challenging question and is worthy of further ad hoc clinical trials. Generally, advances in therapy have led to a significant decline in diabetes-associated complications; however, cardiovascular risk is still a relevant clinical problem. Recently, attention has been devoted to defining a diabetes epigenetic signature which would allow advance in developing new therapeutic options. The results of the present study suggest that histone deacetylase-based therapy could remove one epigenetic tag and change the ROS balance in diabetic patients. Thus, future efforts should be made to clearly assess and correlate the efficacy of epigenetic targeting approaches to the clinical outcomes in T2DM patients.

### Limitations

Study power was originally calculated to detect an effect size of 0.5 on CRP value [17]. The sample size may have therefore been too small to find differences in subgroups. This was probably the case in analyses performed after excluding Placebo arm patients.

We did not measure plasmatic concentrations of resveratrol or its metabolites, meaning that actual substance exposure cannot be determined. Patient compliance could, however, be considered adequate, on the basis of phone calls, capsule counts and the consistent proportional increment in TAS levels with increasing resveratrol doses. The strengths of the present study were the large number of metabolic variables analyzed and the centralized, blindly analyzed, measurements.

## Conclusion

These data suggest that SIRT-1-mediated changes in the epigenome and in the antioxidant response might impact on diabetes-associated risk factors.

## References

[1] Meagher RB, Müssar KJ (2012). The influence of DNA sequence on epigenome-induced pathologies. Epigenetics Chromatin.

[2] Wegner M, Neddermann D, Piorunska-Stolzmann M, Jagodzinski PP (2014). Role of epigenetic mechanisms in the development of chronic complications of diabetes. Diabetes Res Clin Pract.

[3] Das C, Lucia MS, Hansen KC, Tyler JK (2009). CBP/p300-mediated acetylation of histone H3 on lysine 56. Nature.

[4] Tjeertes JV, Miller KM, Jackson SP (2009). Screen for DNA-damage-responsive histone modifications identifies H3K9Ac and H3K56Ac in human cells. EMBO J.

[5] Lo KA, Bauchmann MK, Baumann AP (2011). Genome-wide profiling of H3K56 acetylation and transcription factor binding sites in human adipocytes. PLoS ONE.

[6] Milne JC, Lambert PD, Schenk S (2007). Small molecule activators of SIRT1 as therapeutics for the treatment of type 2 diabetes. Nature.

[7] de Kreutzenberg SV, Ceolotto G, Papparella I (2010). Downregulation of the longevity-associated protein sirtuin 1 in insulin resistance and metabolic syndrome: potential biochemical mechanisms. Diabetes.

[8] Paneni F, Volpe M, Lüscher TF, Cosentino F (2013). SIRT1, p66(Shc), and Set7/9 in vascular hyperglycemic memory: bringing all the strands together. Diabetes.

[9] Togliatto G, Trombetta A, Dentelli P (2015). Unacylated ghrelin induces oxidative stress resistance in a glucose intolerance and peripheral artery disease mouse model by restoring endothelial cell miR-126 expression. Diabetes.

[10] Xia N, Daiber A, Förstermann U, Li H (2017). Antioxidant effects of resveratrol in the cardiovascular system. Br J Pharmacol.

[11] Tomé-Carneiro J, Larrosa M, González-Sarrías A, Tomás-Barberán FA, García-Conesa MA, Espín JC (2013). Resveratrol and clinical trials: the crossroad from in vitro studies to human evidence. Curr Pharmac Des.

[12] Ponzo V, Soldati L, Bo S (2014). Resveratrol: a supplementation for men or for mice?. J Transl Med.

[13] Sahebkar A, Serban C, Ursoniu S (2015). Lack of efficacy of resveratrol on C-reactive protein and selected cardiovascular risk factors—results from a systematic review and meta-analysis of randomized controlled trials. Int J Cardiol.

[14] Sahebkar A, Serban MC, Gluba-Brzόzka A (2016). Lipid-modifying effects of nutraceuticals: an evidence-based approach. Nutrition.

[15] Cicero AFG, Colletti A, Bajraktari G (2017). Lipid lowering nutraceuticals in clinical practice: position paper from an International Lipid Expert Panel. Arch Med Sci.

[16] Bo S, Ponzo V, Ciccone G (2016). Six months of resveratrol supplementation has no measurable effect in type 2 diabetic patients. A randomized, double blind, placebo-controlled trial. Pharmacol Res.

[17] Bo S, Ponzo V, Evangelista A (2017). Effects of 6 months of resveratrol *vs* placebo on pentraxin 3 in patients with type 2 diabetes mellitus. A double-blind randomized-controlled trial. Acta Diabetol.

[18] Trombetta A, Togliatto G, Rosso A (2013). Increase of palmitic acid concentration impairs endothelial progenitor cell and bone marrow-derived progenitor cell bioavailability: role of the STAT5/PPARγ transcriptional complex. Diabetes.

[19] Haigis MC, Guarente LP (2006). Mammalian sirtuins—emerging roles in physiology, aging, and calorie restriction. Genes Dev.

[20] Sosnowska B, Mazidi M, Penson P, Gluba-Brzózka A, Rysz J, Banach M (2017). The sirtuin family members SIRT-1, SIRT3 and SIRT6: their role in vascular biology and atherogenesis. Atherosclerosis.

[21] Vaziri H, Dessain SK, Ng Eaton E (2001). hSIR2(SIRT-1) functions as an NAD-dependent p53 deacetylase. Cell.

[22] Togliatto G, Dentelli P, Brizzi MF (2015). Skewed epigenetics: an alternative therapeutic option for diabetes complications. J Diabetes Res.

[23] Song SB, Jang SY, Kang HT (2017). Modulation of mitochondrial membrane potential and ROS generation by nicotinamide in a manner independent of SIRT1 and mitophagy. Mol Cells.

[24] Rayalam S, Della Fera MA, Baile CA (2011). Synergism between resveratrol and other phytochemicals: implication for obesity and osteoporosis. Mol Nutr Food Res.

[25] Côté CD, Rasmussen BA, Duca FA (2015). Resveratrol activates duodenal SIRT-1 to reverse insulin resistance in rats through a neuronal network. Nat Med.

[26] Cao D, Wang M, Qiu X (2015). Structural basis for allosteric, substrate-dependent stimulation of SIRT-1 activity by resveratrol. Gene Dev.

[27] González-Rodríguez Á, Santamaría B, Mas-Gutierrez JA (2015). Resveratrol treatment restores peripheral insulin sensitivity in diabetic mice in a SIRT-1-indipendent manner. Mol Nutr Food Res.

[28] Sathishkumar C, Prabu P, Balakumar M (2016). Augmentation of histone deacetylase 3 (HDAC3) epigenetic signature at the interface of proinflammation and insulin resistance in patients with type 2 diabetes. Clin Epigenetics.

[29] Yu J, Wu Y, Yang P (2016). High glucose-induced oxidative stress represses sirtuin deacetylase expression and increases histone acetylation leading to neural tube defects. J Neurochem.

[30] Ye X, Li M, Hou T, Gao T, Zhu W, Yang Y (2017). Sirtuins in glucose and lipid metabolism. Oncotarget.

[31] Picard F, Kurtev M, Chung N (2004). SIRT-1 promotes fat mobilization in while adipose tissue by repressing PPAR-γ. Nature.

